# ZFP161 promotes colorectal cancer progression by transcriptionally activating c-MYC

**DOI:** 10.3389/fonc.2025.1680561

**Published:** 2026-02-18

**Authors:** Grania Christyani, Chenghui Cai, Yening Feng, Hyorin Lee, Xinyi Tu, Chao Zhang, Zhenkun Lou, Wootae Kim

**Affiliations:** 1 Department of Integrated Biomedical Science, Soonchunhyang Institute of Medi-bio Science (SIMS), Soonchunhyang University, Cheonan, Chungcheongnam-do, Republic of Korea; 2 College of Biology, Hunan University, Changsha, China; 3 Department of Oncology, Mayo Clinic, Rochester, MN, United States

**Keywords:** c-MYC, *c-MYC* downstream pathway, tumor development, ZFP161, zinc finger protein

## Abstract

Zinc Finger Protein 161 (ZFP161) is a key regulator of Ataxia Telangiectasia and Rad3-related (ATR) signaling, playing a crucial role in maintaining genomic stability. Human ZFP161 activates *c-MYC*, whereas its murine ortholog, ZF5, serves as a putative transcriptional repressor of *c-MYC*. In this study, we identified ZFP161 as a direct regulator of c-MYC. We show that ZFP161 binds to the promoter region of *c-MYC*, modulating its expression and downstream signaling pathways. Additionally, ZFP161 promotes cell proliferation and tumorigenesis through c-MYC regulation and contributes to the malignant transformation of non-cancerous retinal pigment epithelial (RPE-1) cells. Furthermore, high *ZFP161* expression is associated with poor survival in patients with mixed colon adenocarcinoma. These findings suggest that the regulation of *c-MYC* by ZFP161 may represent a potential therapeutic target in *c-MYC*-driven cancers.

## Introduction

Cancer is the second leading cause of death worldwide, with an estimated 9.7 million estimated global deaths annually (1). The most common types of cancer in males are prostate, lung, and colorectal cancer; while in women are breast, colorectal, and lung cancer (2). Cancer is primarily driven by genomic alterations, including point mutations (3), chromosomal translocations (4), and in certain cancers, gene amplifications or deletions of oncogenes and tumor suppressor genes (5). Most oncogenes play important roles in cellular processes, including intracellular signal transduction, cell cycle regulation, growth factor signaling, apoptosis inhibition, and transcriptional regulation (6). Conversely, tumor suppressor genes regulate cell division to restrain uncontrolled cell growth (7), induce apoptosis (8), repair DNA damage, and maintain genomic stability (9), thereby preventing tumorigenesis.

Tumor suppressors or oncogenes are regulated at multiple levels, including genetic, epigenetic, transcriptional, and post-transcriptional mechanisms (10, 11). Aberrant transcription can lead to inappropriate activation of oncogenes or silencing of tumor suppressor genes, contributing to tumorigenesis (12). Transcription is often influenced by genetic mutations, promoter methylation, and most importantly, the activity of transcription factors (12).

Transcription factors are proteins that regulate the transcription of target genes by binding to specific DNA sequences, thus controlling target gene expression (13). The dysfunction of transcription factors significantly contributes to the development of many human cancers. Among eukaryotic transcription factors, zinc finger motifs are highly conserved DNA-binding domains essential for regulating gene expression and play a role in transcriptional repression. These motifs are the most prominent and are typically located at the C-terminus of zinc finger proteins (ZFPs) (14). ZFPs consist of several domains, including the BTB/POZ domain, SCAN domain, Krüppel-associated box (KRAB) domain, SET domain, and C2H2-zinc finger motifs (14).

ZFP161, also known as Zinc Finger and BTB Domain Containing 14 (ZBTB14), is a zinc finger protein composed of 449 amino acid residues. It is characterized by five Krüppel-type zinc finger motifs near the C-terminus and a BTB/POZ domain in the N-terminal region (15). ZFP161 regulates ATR signaling by connecting ssDNA-RPA to the ATRIP/ATR complex, leading to the activation of the ATR signaling pathway during replication stress (16). Gene set enrichment analysis has revealed that ZFP161 targets include multiple transcriptional repressors, such as *RB1, BCL6, TLE2, KLF9*, and *FOXP1* (17). As these factors normally suppress cell cycle progression, their downregulation by ZFP161 may indirectly promote cell proliferation (17). In addition, ZFP161/ZBTB14 has also been found to promote Wnt signaling and play a key role in neural development (18). Furthermore, ZFP161 shares 98% homology with the murine zinc finger protein, ZF5 (19). ZF5 is a putative transcriptional repressor of c-Myc and exhibits growth-suppressive activity in mouse cell lines (20). ZFP161 and its murine ortholog ZF5 function as transcriptional regulators that activate the dopamine transporter (DAT/*SLC6A3*), interleukin-6 (*IL-6*), and leukemia inhibitory factor (*LIF*), while repressing *FMR1* through direct promoter binding (16).

Genetic alterations are major drivers of cancer, affecting the expression and function of oncogenes and tumor suppress (21). These alterations, including mutations, amplifications, and translocations can lead to uncontrolled cell proliferation and resistance to cell death. Among them, one of the most common genetic alterations in human cancer involves the *MYC* proto-oncogenes and its related signaling pathways. MYC activation drives cancer progression through mechanisms that either promote the acquisition of cancer hallmarks intrinsic to tumor cells (22) or influence the tumor microenvironment (TME) and anticancer immune response (23). MYC regulates key processes in cancer cells, including cell growth, differentiation, and metabolism, which are hijacked upon MYC activation to facilitate cellular transformation. Myc expression is closely linked to cell proliferation and cell cycle progression (24). It promotes cell cycle progression by inducing the upregulation of cyclins and CDKs, while downregulating CDK inhibitors such as p15 and p21 (25). Additionally, MYC has been found to enhance mRNA cap methylation, leading to increased protein synthesis and the expression of MYC target genes, thereby promoting cell proliferation (26).

The MYC family includes several genes, such as cellular (*c-MYC*), brain (*B-MYC*), lung (*L-MYC*), neuroblastoma (*N-MYC*), and small (*S-MYC*); however, only *c-MYC*, *L-MYC*, and *N-MYC* have neoplastic potential and are currently used as targets for cancer treatment (27, 28). One of the MYC proteins, c-MYC, is found to be upregulated in 70% of colorectal cancer (CRC) cases (29, 30). Loss of *c-MYC* suppresses intestinal tumorigenesis in mouse models of CRC, indicating that c-Myc function is crucial for tumor development in the mouse colon (31). While c-MYC is important in tumorigenesis and tumor progression, targeting the c-MYC pathway remains challenging due to the absence of an active site for drug binding and its location in the nucleus, which makes it difficult to target with monoclonal antibodies (28, 32).

Despite the importance of ZFP161 in ATR signaling and c-MYC in cancer progression, little is known about the correlation between ZFP161 and c-MYC in CRC. Our study identifies ZFP161 as a novel regulator of c-MYC expression, providing new insights into the regulation of its activity. We demonstrate that ZFP161 regulates transcription by binding to the *c-MYC* promoter, thereby modulating its expression and downstream signaling pathways. Additionally, we found that ZFP161 promotes cell proliferation and tumorigenesis through the regulation of *c-MYC*. Collectively, our research suggests that ZFP161 regulates c-MYC expression, highlighting the potential of ZFP161 levels as a predictive marker for poor survival in colorectal cancer.

## Materials and methods

### Cell culture

All cell lines used were purchased from American Type Culture Collection (ATCC). HEK293T (CRL-3216), and hTERT-RPE-1 were cultured in Dulbecco’s Modified Eagle’s Medium, while HCT116 (CCL-247) were maintained in McCoy’s 5A medium. All media were supplemented with 10% fetal bovine serum (FBS), and the cells were cultured at 37 °C in 5% (v/v)CO^2^.

### Plasmids and antibodies

pCMV6–Myc-ZFP161 vector was purchased from ORIGENE. ZFP161 was sub-cloned into pCMV and pLVX3 vectors. The following antibodies were used: anti-ZFP161 (Santa Cruz (C-4): sc-514298, 1:1000), anti-GAPDH (Proteintech: 60004-1-lg, 1:10,000), anti-β-actin (Sigma: A2228, 1:2000), and anti-c-MYC (Santa Cruz: sc-40, 1: 1000, sc764, 1:1000).

shRNAs and sgRNAs All ZFP161 shRNAs were purchased from Sigma. ZFP161 shRNA #1: 5’-TTGATAGTTCTTCGGTCATAG-3’, ZFP161 shRNA #2: 5’-GACATGAAGTTTGAGTATTTG-3’. ZFP161 knockout HCT116 cells were generated using CRISPR. Briefly, two ZFP161 sgRNAs, 5’-CACCGGGGAAGACGTTTTCTGATGA-3’ and 5’-CACCGGGCAGGCAATCTGCTCCCGA-3’, were inserted into the LentiCRISPR v2 vector (Addgene). These shRNA and sgRNA vectors were used for lentiviral infection.

### DNA transfection, virus packaging, and lentiviral infection

All DNA transfections were conducted using TransIT-X2 (MIRUS Bio). To infect HCT116 cells, lentiviruses were produced in HEK293T cells with specific shRNA, sgRNA, pMD2.G, and pSPAX2. The media containing the lentivirus was collected 48 hours post-transfection. The viral supernatant was filtered through a 0.45 µm membrane to remove HEK293T cell debris and supplemented with 8 µg/ml polybrene to improve infection efficiency, prior to infecting HCT116 cells.

### Western Blot

Cells were lysed (20 mM Tris-HCl, pH 8.0, 100 mM NaCl, 1 mM EDTA, 0.5% Nonidet P-40 with protease inhibitors) and centrifugated at 14,000 × g for 15 min. Concentration of the supernatant was measured. Samples were mixed with 2x sodium dodecyl sulfate (SDS) loading buffer at a 1:1 ratio and boiled. Proteins were separated by SDS-PAGE and transferred onto PVDF membrane. The membrane was then immunoblotted with indicated antibodies.

### Quantitative PCR

RNA was isolated with TRIzol RNA Isolation Reagents (Thermo Fisher). Reverse transcription was performed with PrimeScript™ RT Reagent Kit (Takara), and quantitative PCR was performed with Power SYBR Green Master Mix (Thermo Fisher). Gene expression levels were calculated using the 2^−ΔΔCt^ method.

### Dual luciferase reporter assays

Thermo Renilla-Firefly Luciferase Dual Assay Kit (#16185) was used to perform dual luciferase reporter assays which allow simultaneous measurement of both firefly and Renilla luciferases from the same sample. The cells were transfected with equal molar ratios of firefly and Renilla luciferase reporter constructs and incubated for 16–72 hours at 37 °C. Cells were rinsed with PBS buffer and then lysed using the Cell Lysis Buffer (100ul/well) on a platform shaker for 15 minutes. This was followed by Luciferase Dual Assay. The luminometer and injector were prepared and the system was primed with working solution. 10-20µL of cell lysate were added to a black flat-bottomed 96-well plate. Using the luminometer’s injectors, working solution (50µL/well) was injected into the wells. Luminometer were programmed to immediately detect the light output using a 640nm LP filter to capture the red firefly luciferase signal. Then, the sample was immediately read using a 525 ± 20nm BP filter to capture the green Renilla luciferase signal. The steps were repeated for all samples in the plate. Firefly luciferase activity was normalized to Renilla luciferase activity to control for transfection efficiency (33, 34).

### Chromatin immunoprecipitation (ChIP) assays

ChIP was conducted using the Magna Chip (#17-10461) following the manufacturer’s detailed protocol. The assay began with the cross-linking of protein-DNA complexes in cultured cells using formaldehyde at a final concentration of 1% for 10 minutes at room temperature. The reaction was quenched with glycine by adding 2ml of 10X Glycine to each dish to quench unreacted formaldehyde for 5 minutes. Cells were then lysed with Cell Lysis Buffer, and chromatin was sheared to an average length of 100–200 base pairs using sonication. The sheared chromatin was incubated overnight at 4 °C with specific antibodies pre-bound to magnetic beads provided in the kit. After binding, the bead-bound chromatin was washed multiple times to remove non-specifically bound material. The protein-DNA complexes were then eluted from the beads and the cross-links reversed by incubating at 65 °C overnight. DNA was purified using the purification materials included in the kit. Quantitative PCR was subsequently performed to quantify the enrichment of specific DNA sequences (35, 36). The primers used for the *c-MYC* promoter were: forward 5’- CTCAGTCTGGGTGGAAGGTATC-3’ and reverse 5’-CAGAGCGTGGGATGTTAGTGTA-3’.

### Colony formation assay

500 cells were plated in triplicate in each well of 6 well plates. Cells were incubated for 10–14 days at 37 °C to allow colony formation. Colonies were stained with Giemsa and counted.

### Soft agar assay

The soft agar colony formation assay was performed as described previously (37, 38). In brief, RPE-1 cells were transduced with pLVX3-ZFP161 or pLVX3 empty vectors control viruses. For the base layer, 1.2% agarose in complete DMEM supplemented with 10% fetal bovine serum (FBS) was prepared and added to 35-mm dishes. Cells were suspended in 0.3% agarose prepared in complete DMEM containing 10% FBS and plated onto the solidified base layer. Cells were cultured for 2 weeks at 37°C in 5% CO_2_. Colonies were counted under a light microscope (ECLIPSE 80i; Nikon). Images were captured (SPOT 2 Megasample; Diagnostic Instruments, Inc.) and processed using SPOT software (version 4.6; Diagnostic Instruments, Inc.).

### Cell viability assay

Cells were seeded into 96-well plates (1,000 cells/well) and allowed to adhere for 24 hours, after which cell viability was measured at the indicated time points. Cell viability was assessed with CellTilter 96 AQueous One Solution Cell Proliferation Assay (MTS) according to the manufacturer’s instructions (Promega). Briefly, 20µl of MTS was added to each well and the plates were incubated at 37 °C for 3 hours. The absorbance was then measured with a microplate reader at 490nm (Tecan Infinite M1000 PRO).

### Statistics and reproducibility

All statistical analysis was performed using Prism v.8.0 (GraphPad) or R studio. Data in bar and line graphs are presented as mean ± S.D. of at least three independent experiments, performed using independently prepared biological samples. All western blot assays shown here were successfully repeated at least three times. Unless otherwise noted, statistical comparisons between two groups were performed using two-tailed Student’s t-tests, whereas comparisons among multiple groups were analyzed using one-way Analysis of Variance (ANOVA). A p-value of < 0.05 was considered statistically significant.

## Results

### *ZFP161* positively correlates with *c-MYC* expression

**Figure 1:**
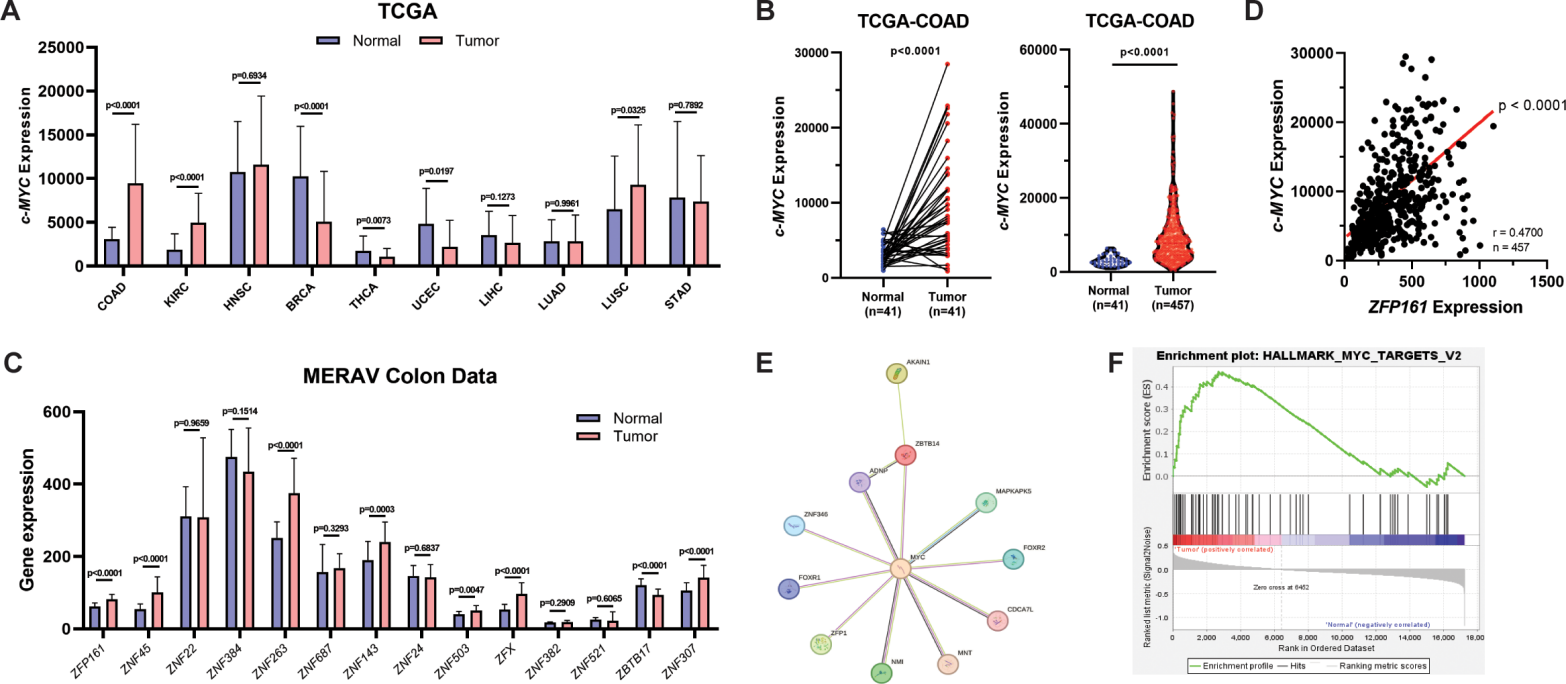
*ZFP161* positively correlates with *c-MYC* expression. **(A)***c-MYC* expression in various cancers based on TCGA data. **(B)***c-MYC* expression in colon cancer. Paired tumor and adjacent normal tissues from the same patients analyzed with paired t-test (left). Tumor and normal groups with different sample sizes analyzed with unpaired two-tailed t-test (right). **(C)** Comparison of zinc-finger gene expression between normal and tumor samples in colon cancer derived from the MERAV database analyzed with unpaired two-tailed t-test. **(D)** Correlation between ZFP161 and *c-MYC* expression analyzed by Pearson correlation (r = 0.47, 95% CI = 0.3953–0.5385, p<0.0001, n=457). **(E)** Predicted protein–protein interaction network between ZFP161 (ZBTB14) and *c-MYC* generated by STRING. **(F)** Gene set enrichment analysis (GSEA) of ZFP161-associated genes.

Given that *c-MYC* is highly expressed in cancer, we performed a *c-MYC* expression screening using the TCGA database (**Figure 1A**). TCGA-COAD data confirmed increased *c-MYC* expression in colon tumors, both in paired normal-tumor patients (**Figure 1B**, left) and unpaired samples (**Figure 1B**, right).

Since ZFP161 (also known as ZF5) is a known negative regulator of *c-Myc* in mice (16), we sought to determine whether this role is conserved in humans. Unlike in mice, where ZFP161 functions as a negative regulator of *c-Myc*, in human colon cancer, ZFP161 levels were significantly higher compared to normal cells (**Figure 1C**). This suggests that ZFP161 may have a distinct regulatory role in human cancers. Consistently, TCGA-COAD showed a significant positive correlation between *ZFP161* and *c-MYC* expression (**Figure 1D**).

To further validate this association at the protein level, we examined protein-protein interactions using the STRING database (https://string-db.org/). STRING analysis annotated a direct interaction between ZFP161 (ZBTB14) and *c-MYC* (**Figure 1E**). Additionally, Gene Set Enrichment Analysis (GSEA) of *ZFP161* revealed that it is functionally linked to the hallmark *c-MYC* target signature (**Figure 1F**), suggesting that ZFP161 may play a role in *c-MYC* regulation.

Taken together, these data demonstrate that *ZFP161* is positively correlated with *c-MYC* expression and may contribute to *c-MYC*-driven oncogenic pathways.

### ZFP161 positively regulates *c-MYC* mRNA level

**Figure 2:**
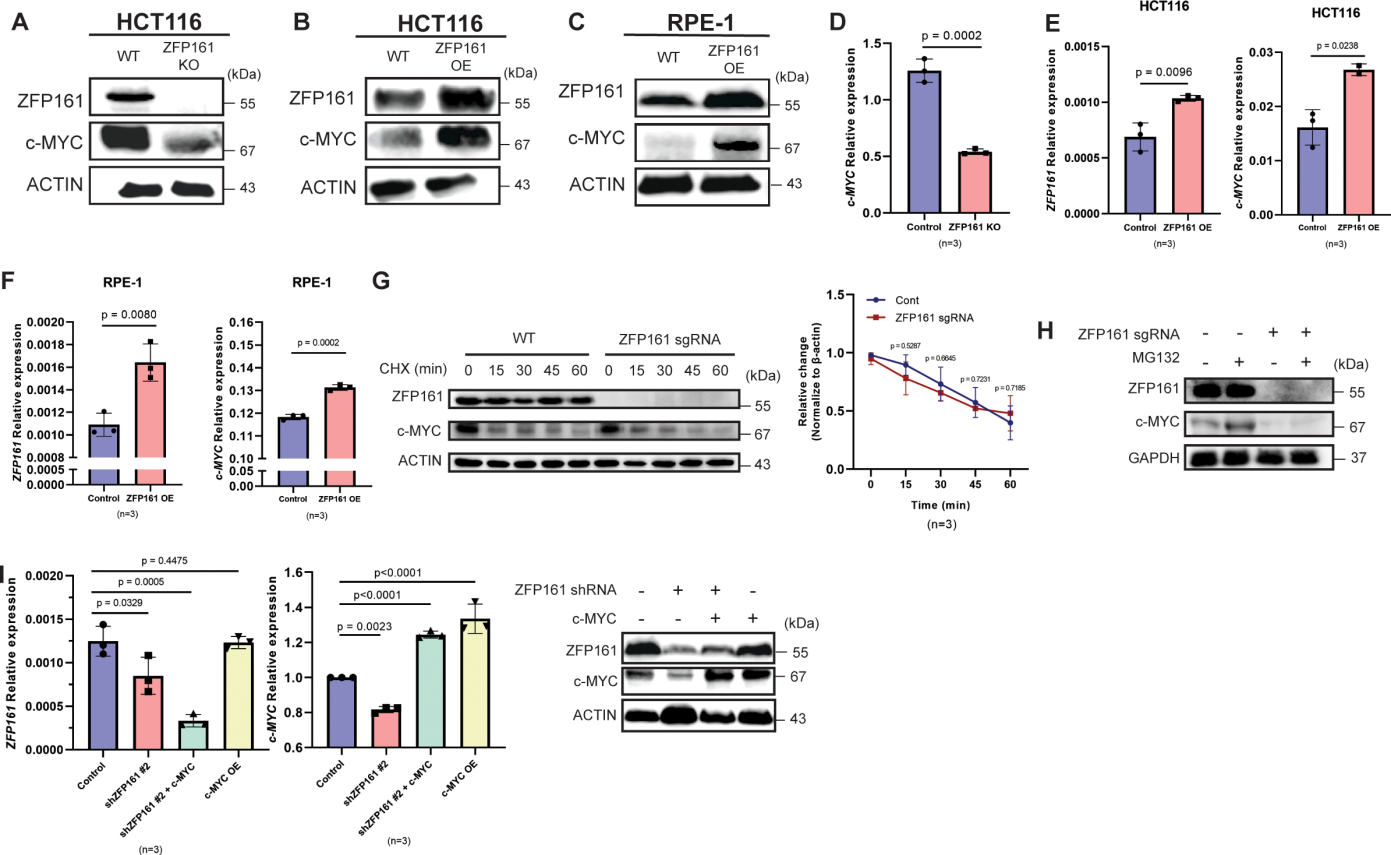
ZFP161 positively regulates *c-MYC* mRNA level. **(A)** Low expression of *c-MYC* in ZFP161 KO cells. **(B, C)** Increased *c-MYC* protein levels upon ZFP161 overexpression in HCT116 **(B)** and RPE-1 **(C)** cells detected by western blot. **(D)** Expression of *c-MYC* mRNA level in ZFP161 KO cells. **(E, F)**. Increased *c-MYC* mRNA levels upon ZFP161 overexpression in HCT116 **(E)** and RPE-1 **(F)** cells measured by RT-qPCR. **(G, H)**. Cells were treated with 100uM CHX for the indicated time points **(G)** and 20 µM MG132 for 4 hr **(H)**. Each sample was immunoblotted with indicated antibodies. **(I)** mRNA level of *ZFP161* (left) and *c-MYC* (middle) upon ZFP161 KD measured by RT-qPCR and *c-MYC* overexpression upon ZFP161 KD measured by western blot (right). The graphs represent mean ± S.D. of n = 3 independent experiments, two-tailed, paired t-test.

To investigate the role of ZFP161 in c-MYC regulation, we generated ZFP161 knockout and knockdown HCT116 colon cancer cells and validated the ZFP161 protein levels using Western blot. The c-MYC level was clearly reduced in ZFP161-deficient cells (**Figure 2A**, **Supplementary Figure 1A**), supporting the hypothesis that ZFP161 enhances c-MYC expression. Consistently, *ZFP161* overexpression increased *c-MYC* protein levels in both HCT116 (**Figure 2B**) and RPE-1 cells (**Figure 2C**), implying that ZFP161 enhances c-MYC expression. To determine whether ZFP161 regulates c-MYC protein stability, we performed a cycloheximide (CHX) chase assay and observed that *ZFP161* depletion did not significantly alter the c-MYC degradation rate (**Figure 2G**), suggesting that ZFP161 primarily regulates the expression of *c-MYC* at the transcriptional level, rather than its stability. To confirm whether this regulation occurs at the transcriptional level, we analyzed *c-MYC* mRNA expression via qPCR. We found that *c-MYC* mRNA levels were significantly decreased in *ZFP161*-deficient cells (**Figure 2D**, **Supplementary Figure 1B**). Conversely, ZFP161 overexpression led to increased *c-MYC* mRNA levels in both HCT116 cancer cells (**Figure 2E**) and non-malignant RPE-1 cells (**Figure 2F**). This suggests that ZFP161 regulates c-MYC expression at the transcriptional level.

To further confirm that ZFP161 primarily regulates *c-MYC* expression and not its protein stability, we performed an MG132 assay to inhibit proteasomal degradation (**Figure 2H**). Notably, treatment with the proteasome inhibitor MG132 failed to rescue the reduced c-MYC protein levels in ZFP161-deficient cells, confirming that ZFP161 functions via transcriptional regulation.

Because ZFP161 strongly regulates *c-MYC* expression, we next tested whether c-MYC, in turn, regulates *ZFP161*. qPCR analysis revealed that c-MYC overexpression to *ZFP161* knockdown cells further decreased *ZFP161* mRNA levels (**Figure 2I**). Furthermore, c-MYC overexpression restored MYC protein levels in *ZFP161*-depleted cells.

### ZFP161 binds directly with the promoter region of *c-MYC* and regulates c-MYC expression

**Figure 3:**
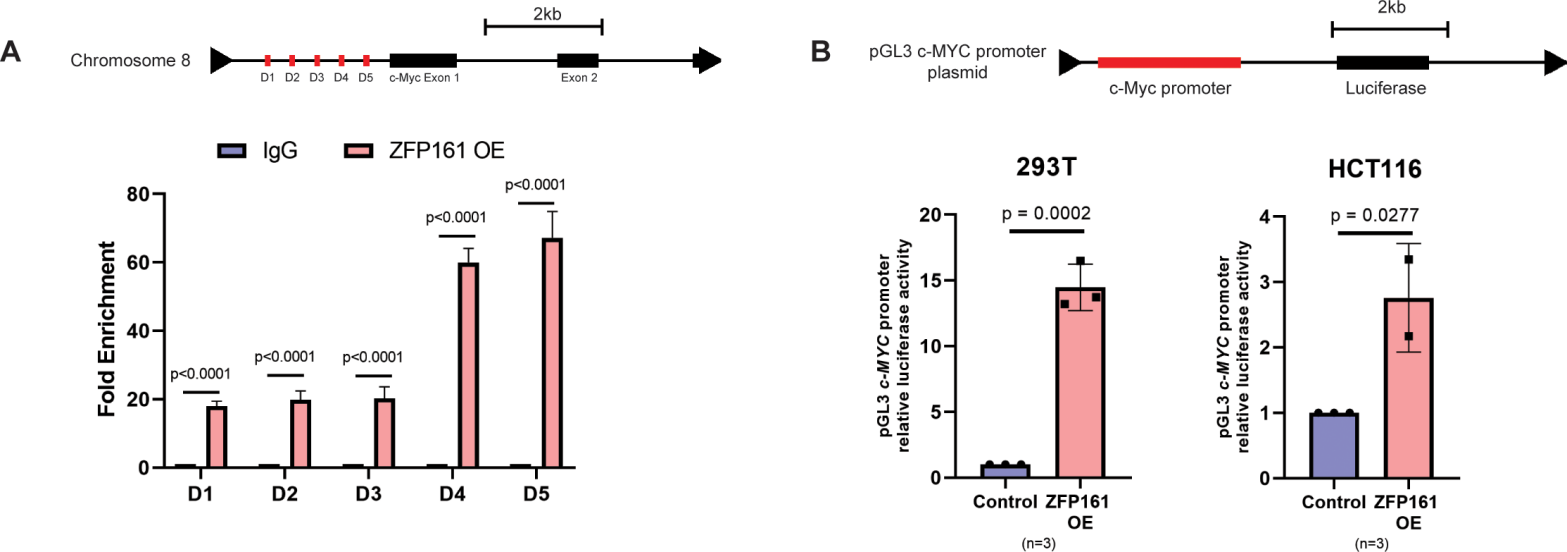
ZFP161 binds directly with the promoter region of *c-MYC* oncogene and regulates *c-MYC* expression. **(A)** Schematic diagram of the human *c-MYC* promoter region indicating the locations of five primer pairs used for ChIP-qPCR analysis (upper panel). ChIP-qPCR analysis showing ZFP161 enrichment at the indicated regions of the *c-MYC* promoter; IgG was used as a negative control (lower panel). **(B)** Relative luciferase activity of the MYC promoter reporter in ZFP161-overexpressing cells measured by dual-luciferase reporter assay. Independent replicates of cell lines (n=3) were analyzed using an unpaired, two-tailed Student's t-test.

Since our results indicated that ZFP161 regulates *c-MYC* transcription, we next investigated whether ZFP161 directly binds to the *c-MYC* promoter. Previous reports identified five main genomic fragments within the *c-MYC* promoter region that are important for regulation (33). To assess the interaction between ZFP161 and the *c-MYC* promoter, we performed a chromatin immunoprecipitation (ChIP) assay, which confirmed the binding of ZFP161 to chromatin fragments containing the *c-MYC* promoter region. Notably, ZFP161 was enriched in the D4-D5 region (168 bp) (**Figure 3A**). The nearby regions (D1–D3) were also enriched, though less significantly than the D4–D5 region.

To further determine whether the *c-MYC* promoter region drives ZFP161-dependent transcriptional activity, we conducted a luciferase reporter assay. The results showed a significant increase in *c-MYC* promoter luciferase activity in both HCT116 and 293T cells upon ZFP161 overexpression (**Figure 3B**). Taken together, these findings suggest that ZFP161 contributes to the transcriptional regulation of *c-MYC* in human cell.

### ZFP161 promotes cell proliferation and tumorigenesis through the *c-MYC* downstream pathway

**Figure 4:**
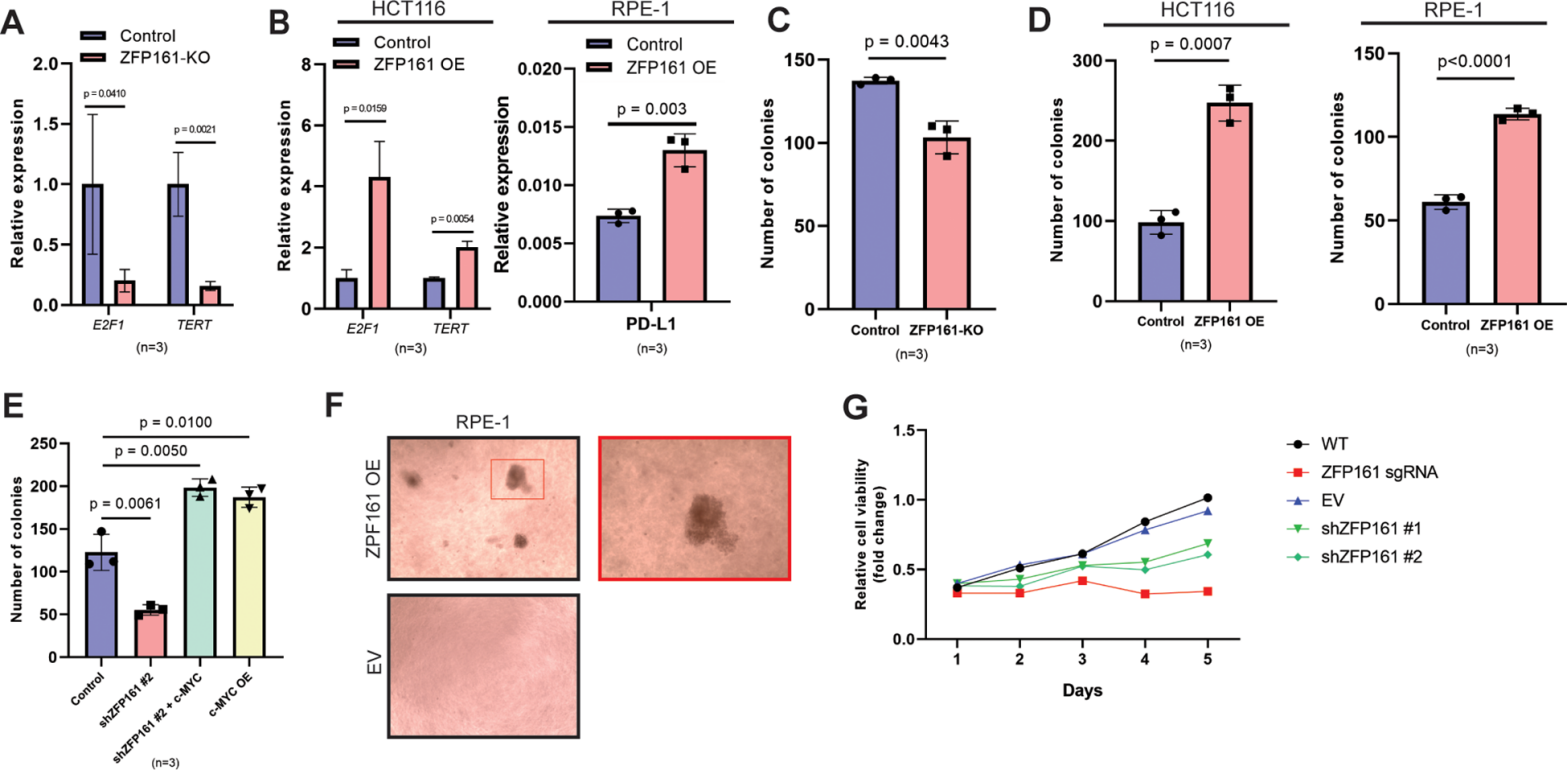
*ZFP161* promotes cell proliferation and tumorigenesis through *c-MYC* downstream pathway. **(A)** mRNA levels of *E2F1* and *TERT* in ZFP161 KO cell. **(B)** Relative mRNA levels of *E2F1* and *TERT* in HCT116 cells (left) and PD-L1 in RPE-1 cells (right) upon ZFP161 overexpression (ZFP161 OE), compared to the control group. **(C)** Colony formation assay of HCT116 ZFP161 knockout cells; colonies were counted after 14 days. **(D)** ZFP161 overexpression increases colony number in both HCT116 (left) and RPE-1 (right) **(E)** ZFP161 knockdown reduces colony formation and is rescued by c-MYC overexpression. Control cells, ZFP161 knockdown cells (shZFP161 #2), ZFP161 knockdown cells with c-MYC overexpression (shZFP161 #2 + c-MYC), and c-MYC overexpressing cells (c-MYC OE) were analyzed. **(F)** Soft agar assay. RPE-1 cells (10,000 cells/well) were seeded on a plate with an agarose layer and the colony number was counted after 14 days. **(G)** HCT116 were plated in 96 well plates (1,000 cells/well) were seeded and cell viability was assessed. The graphs represent mean ± S.D. of n = 3 independent experiments. Statistical analysis was performed using unpaired Student’s t-test for panels **(A–D)** and F, one-way ANOVA for panel **(E)**, and two-way ANOVA for panel **(G)**.

Since ZFP161 regulates c-MYC protein levels, we hypothesized that modulation of c-MYC by ZFP161 would affect *c-MYC* downstream genes. Specifically, we investigated the impact of ZFP161 the expression of key *c-MYC* target genes, such as *E2F1*, *TERT*, and PDL1. *E2F1* is required for the G1/S transition in the cell cycle (25). Telomerase reverse transcriptase (*TERT*), a component of telomerase, is contributing to telomerase maintenance and cellular immortality (39). Additionally, *c-MYC* promotes tumor immune evasion by regulating the expression of immune checkpoints, including Programmed Death-Ligand 1 (PD-L1) (40). Our results showed that *ZFP161* knockout (KO) cells exhibited reduced *E2F1* and *TERT* mRNA levels (**Figure 4A**). In contrast, *ZFP161* overexpression significantly increased *E2F1* and *TERT* mRNA levels in HCT116 cells as well as PD-L1 in RPE-1 cells (**Figure 4B**). These findings suggest that ZFP161 modulates *c-MYC* downstream pathways by regulating c-MYC expression.

Previous studies have shown that *c-MYC* Box I and II promote stem cell generation and are crucial for *c-MYC*-mediated colony formation and transformation of normal cells into cancer cells (34). Since ZFP161 regulates *c-MYC* downstream genes, we next studied the effect of ZFP161 on cell proliferation and tumorigenesis using a colony formation assay. Strikingly, ZFP161 deficiency reduced the number of colonies (**Figure 4C**, **Supplementary Figure 2A**), indicating that ZFP161 is involved in cell proliferation.

Consistently, ZFP161 overexpression significantly increased the number of colonies in both HCT116 and RPE-1 cells (**Figure 4D**). Furthermore, c-MYC overexpression restored the colony-forming ability in ZFP161-depleted cells, indicating that the loss of ZFP161 can be compensated by *c-MYC* (**Figure 4E**). However, this observation does not exclude the possibility that ZFP161 also exerts function independent of c-MYC.

Since c-MYC is known to promote anchorage-independent growth, we performed a soft agar assay using non-malignant RPE-1 cells (38). Notably, ZFP161-overexpressing RPE-1 cells exhibited the ability to form colonies in soft agar (**Figure 4F**). This indicates that ZFP161 enhances cell proliferation, confers anchorage-independent growth, and malignant transformation activity of RPE-1 cells.

Finally, we assessed the viability of ZFP161-depleted cells and observed a significant reduction in cell viability in ZFP161 knockout cells compared to wild-type and empty vector controls (**Figure 4G**). ZFP161 knockdown also led to a decrease in cell viability, although this reduction was less pronounced than that seen in ZFP161 knockout cells. These results collectively suggest that ZFP161 promotes cell proliferation and tumorigenesis through modulation of the *c-MYC* downstream pathway.

### ZFP161 and c-MYC are potential markers for poor survival in colorectal cancer

**Figure 5:**
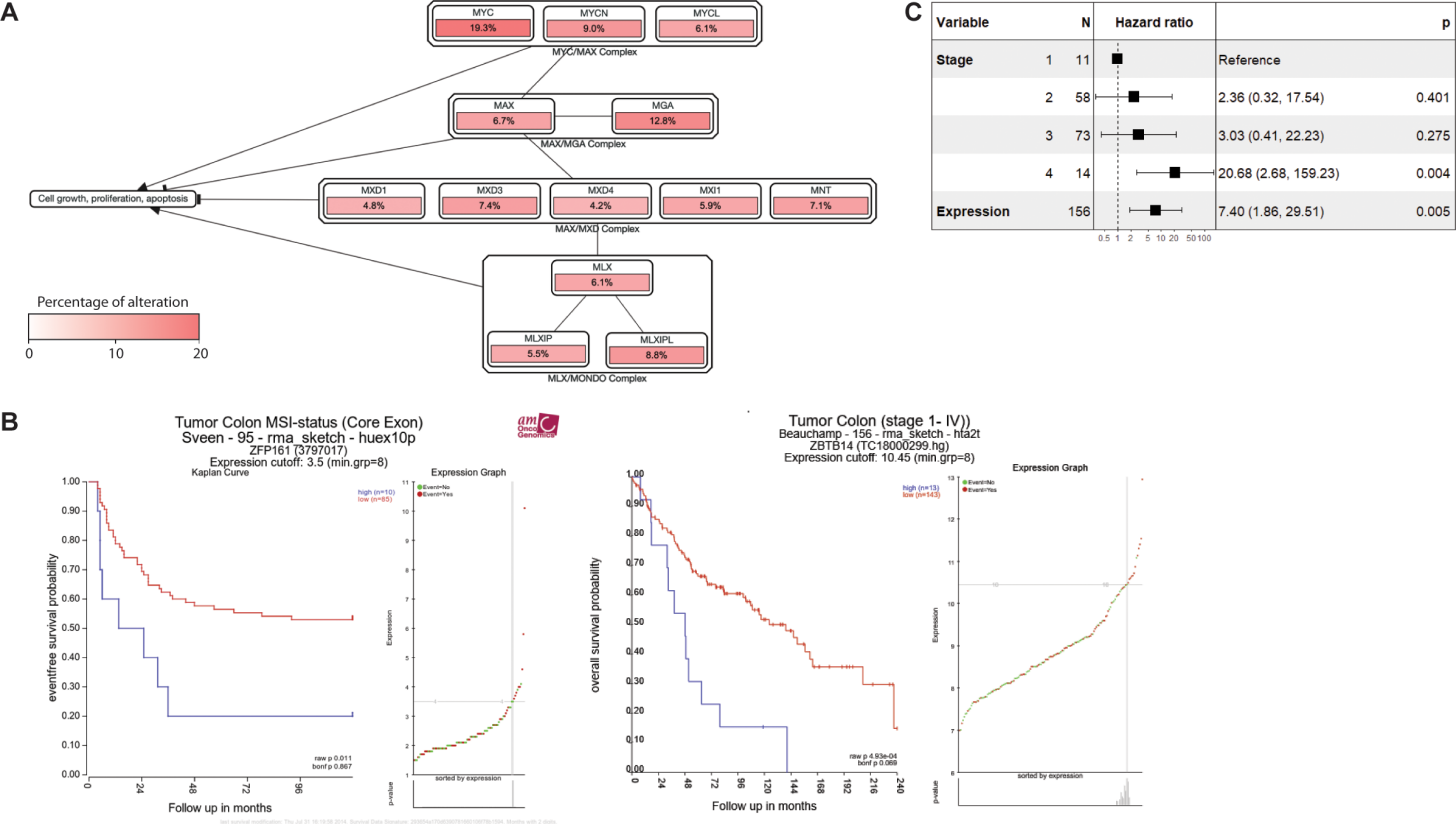
ZFP161 and c-MYC is a potential marker for poor survival in colorectal cancer. **(A)** cBioPortal Pathway Mapper Analysis of gene expression alteration using a white-to-red color scale. **(B)** Kaplan-Meier survival plots were generated based on two different datasets from the R2 website, showing the expression of *ZBTB14/ZFP161* in mixed colon adenocarcinoma patients. **(C)** Forest plot of multivariable Cox proportional hazards regression for overall survival. Clinical stage and *ZFP161* expression were included as covariates. Hazard ratios (squares) with 95% confidence intervals (horizontal lines) are shown. The model included 156 patients, with 78 death events recorded. Stage I was used as the reference category. Stage IV patients had significantly worse survival (HR = 20.68, p = 0.004). Elevated ZFP161 expression was independently associated with poor prognosis (HR = 7.40, p = 0.005).

Our results show that both ZFP161 and c-MYC play roles in cancer progression. To assess the clinical relevance of ZFP161 and c-MYC, we analyzed genetic alteration data from TCGA and visualized the frequency of alterations in MYC-related complexes. **Figure 5A** reveals the genetic alteration frequencies of key components within MYC-related complexes, analyzed from The Cancer Genome Atlas (TCGA) data. Notably, in the critical MYC/MAX heterodimer complex, *c-MYC* exhibits the highest alteration rate at 19.3%, significantly surpassing *MYCN* (9.0%) and *MYCL* (6.1%). This predominant alteration of *c-MYC* underscores its central role as a frequently dysregulated oncogene in cancer. Beyond this, other MYC-associated complexes, including MAX/MGA (*MAX* 6.7%, *MGA* 12.8%), MAX/MXD (*MXD1* 4.8% - *MNT* 7.1%), and MLX/MONDO (*MLX* 6.1%, *MLXIP* 5.5%, *MLXIPL* 8.8%), also show varying degrees of genetic alteration, indicating broader dysregulation within the MYC signaling network.

The MYC/MAX heterodimer complex function as transcriptional activator, where the binding of MYC/MAX heterodimer to E-box DNA can activate transcription of genes involved in cell growth, metabolism, and proliferation (41). Alterations in this complex can lead to persistent activation of MYC target genes, driving uncontrolled cell division and tumorigenesis. This suggests that *c-MYC* is a key player in cancer-associated genetic changes and may serve as a critical driver in tumorigenesis.

Furthermore, Kaplan-Meier survival analysis illustrated that high *ZFP161* expression was associated with poorer survival in patients with mixed colon adenocarcinoma (**Figure 5B**). In addition, we performed multivariable Cox proportional hazards regression analysis to investigate whether *ZFP161* expression and clinical stage were associated with overall survival in colorectal cancer patients (**Figure 5C**). Stage IV patients were strongly correlated with increased risk of death (HR = 20.68, 95% CI: 2.68–159.23, p = 0.004), whereas Stage II and Stage III patients showed a trend toward increased risk but did not reach statistical significance. Importantly, high ZFP161 expression was an independent prognostic factor, associated with a 7.40-fold higher hazard of death (95% CI: 1.86–29.51, p = 0.005). The concordance index of the model was 0.691, indicating good predictive performance. Collectively, these results suggest that the co-expression of ZFP161 and c-MYC may serve as a potential marker for poor prognosis in colon cancer.

## Discussion

Previous study has shown that ZFP161 is a regulator of genomic stability through the ATR/ATRIP complex recruitment (16). Utilizing ZFP161 knockout mice, ZFP161 expression is shown to be significantly lower in tumors and loss of ZFP161 leads to higher chromosomal instability, the formation of micronucleus normochromic erythrocytes, and an increase in γH2AX positive cells (16).

In this study, we identified the novel role of ZFP161 as a regulator of *c-MYC* expression and function. We found that ZFP161 binds directly with the promoter region of the *c-MYC* oncogene, which explains its colocalization in the cellular nuclear of HCT116 and RPE-1 cells. ZFP161 and c-MYC expression are positively correlated and any changes in ZFP161 expression also led to alteration of c-MYC downstream pathway. Moreover, c-MYC overexpression can compensate for the effect of ZFP161 loss. ZFP161 was also found to promote cell proliferation and tumorigenesis through the regulation of c-MYC. Through this study, we demonstrate a novel connection of ZFP161 and c-MYC compared to prior reports, highlighting the role of ZFP161 in c-MYC–driven tumorigenesis.

Our findings suggest that ZFP161 serving as a positive regulator in human colorectal cancer cells, while its ortholog in mice, ZF5, functions as a transcriptional repressor of *c-Myc*. This may be attributed to several factors, including species-specific divergence, as the transcriptional regulation diverges significantly between humans and mice (42). Another factor includes the cellular environment of cancerous cells compared to normal cells. Certain genes, such as TGF-β, inhibit proliferation in normal cells or during the early stages of tumorigenesis but promote tumorigenesis in advanced cancer (43). In addition, the interaction partner in mice and human might be different, leading to different function between species (44). This explains the different functions of ZFP161 in human colorectal cancer cells versus mouse models.

c-MYC overexpression is found in approximately 70% of human colorectal cancers (45). *c-MYC* gene amplification is significantly more frequent in metastases than in primary tumors. *c-MYC* expression induces metabolic changes early in carcinogenesis and regulates genes involved in mitochondrial function and DNA methylation (45). *c-MYC* knockdown reversed these metabolic changes and inhibited cell growth, highlighting potential therapeutic targets (46). Higher *c-MYC* expression correlates with poorer progression-free and overall survival and resistance to anti-EGFR therapies in metastatic colorectal cancer (47).

Although targeting c-MYC can promote tumor regression, therapy targeting c-MYC has not yet been successfully established. Current early-stage clinical trials are exploring c-MYC inhibitors that either suppress c-MYC expression, inhibit c-MYC protein biosynthesis, or target c-MYC for proteasomal degradation. c-MYC can also be indirectly inhibited by targeting synthetic lethal interactions, including HDACs, CDK4, CDK6, CDK7, and CDK9, as well as DNA repair genes such as *CHK1* and *ATR* and anti-apoptotic genes (22). Despite promising preclinical results, most inhibitors have not yet entered clinical trials. Antisense RNA oligonucleotides as a therapeutic strategy have been tested in phase I and II clinical trials, yet most of them are terminated (48). To date, OMO-103 is the only c-MYC-targeting that is currently being tested in clinical trials. Dose-escalation Phase I study in patients with various solid tumors confirms the safety of OMO-103 (49).

Our finding shows that *ZFP161* expression was significantly elevated in the stage IV patients (**Figure 5C**). This raises the possibility of ZFP161 being explored in future studies as a biomarker for risk stratification in colorectal cancer. In addition, STRING analysis indicated that ZFP161 may interact indirectly with histone-modifying enzymes, such as lysine methyltransferases, suggesting a potential role in chromatin regulation. Future studies examining these interactions will provide further insight.

A limitation of our study is that patient age which is a prognostic factor in cancer outcomes, was not available in the Beauchamp et al. dataset and therefore could not be incorporated into our multivariate analysis. Future studies using larger cohorts with more comprehensive clinical annotations will be necessary to validate our findings.

Further investigation using ZFP161 knockout cells and promoter activity assays following ZFP161 depletion will be essential to establish the direct dependence of the *c-MYC* promoter on ZFP161. Moreover, validation at the protein level of the observed changes in *E2F1, TERT*, and *PD-L1* expression would be important for future studies. Additional research will be needed to determine whether ZFP161 overexpression is sufficient to drive tumor formation in animal models.

In summary, our study confirms the association between ZFP161 and c-MYC in CRC. ZFP161 regulates c-MYC and its downstream pathway, leading to cell proliferation and tumorigenesis, highlighting another potential target for therapeutic intervention in c-MYC-driven cancers.

## Data Availability

The data supporting the conclusions of this article are included within the article and its Supplementary Figures.

## Supplementary material

Supplementary Figure 1 Expression of c-MYC and ZFP161 in ZFP161 KD cells. **(A)** Protein levels of c-MYC in ZFP161 knockdown cells detected by western blot. **(B)** mRNA levels of *ZFP161* and *c-MYC* in ZFP161 knockdown cells measured by RT-qPCR.

Supplementary Figure 2 Colony Formation of ZFP161 KD cells. A. Colony formation assay of HCT116 ZFP161 knockdown cells; colonies were counted after 14 days.
