# Supplementary figures and images for: ZFP161 promotes colorectal cancer progression by transcriptionally activating c-MYC
Source: Front Oncol. 2026 Feb 18;15:1680561. doi: 10.3389/fonc.2025.1680561 (PMC12956533; doi:10.3389/fonc.2025.1680561)

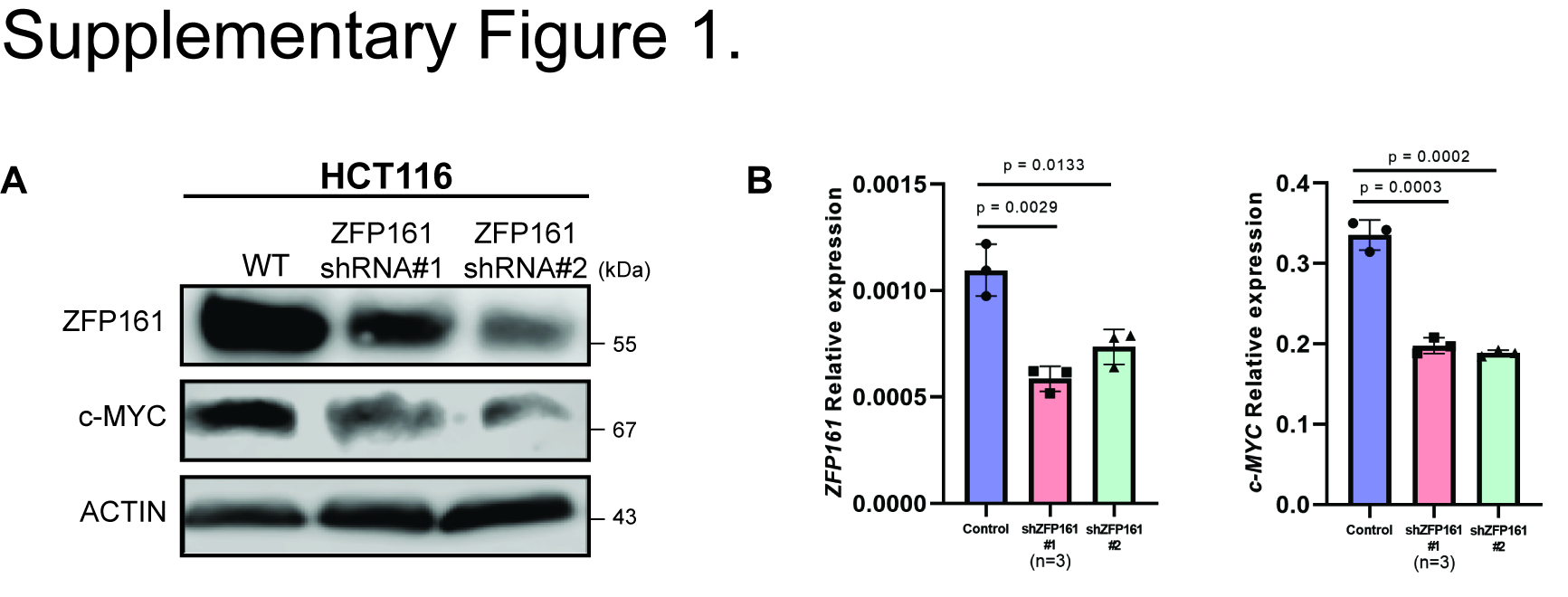

Supplement: Supplementary Figure 1 — Expression of c-MYC and ZFP161 in ZFP161 KD cells. (A) Protein levels of c-MYC in ZFP161 knockdown cells detected by western blot. (B) mRNA levels of ZFP161 and c-MYC in ZFP161 knockdown cells measured by RT-qPCR. [file Image1.tif]

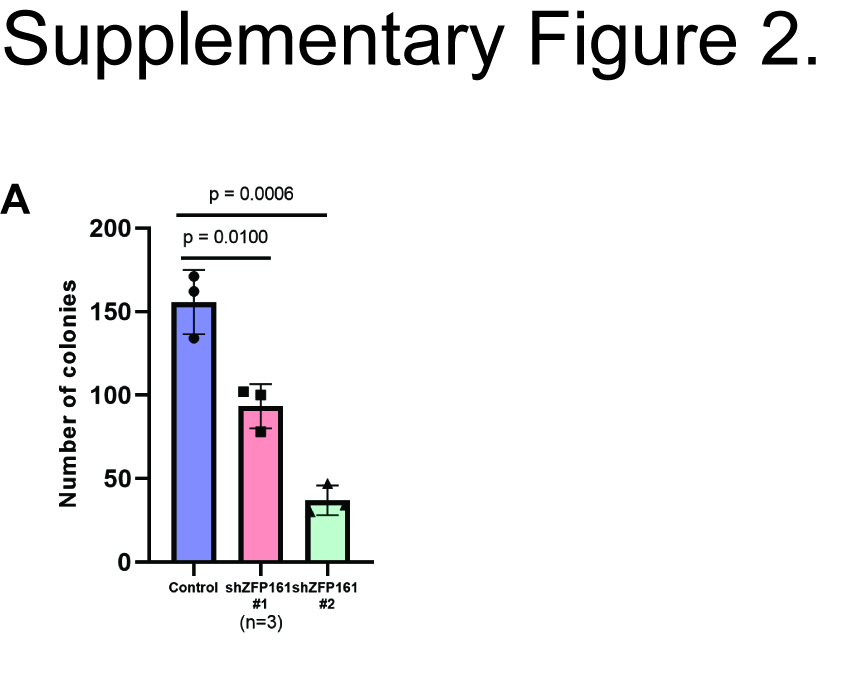

Supplement: Supplementary Figure 2 — Colony Formation of ZFP161 KD cells. A. Colony formation assay of HCT116 ZFP161 knockdown cells; colonies were counted after 14 days. [file Image2.tif]
